# Burnout and depression in first-year medical students across the academic year in the United States

**DOI:** 10.1007/s44192-025-00175-9

**Published:** 2025-04-01

**Authors:** Jonathan Shaw, Charles Lai, Peter Bota, Deborah Wright

**Affiliations:** School of Medicine, California University of Science and Medicine in Colton, 1501 Violet St, Colton, CA 92324 USA

**Keywords:** Medical education, Psychology, Mental illness

## Abstract

**Objective:**

This study examined the prevalence and severity of burnout and depression in first-year medical students at different points in the academic year.

**Methods:**

U.S. first-year allopathic medical students with a pass/fail preclinical curriculum were emailed through the school’s email list with four rounds of surveys consisting of the full set of questions from the Copenhagen Burnout Inventory and the Patient Health Questionnaire-9, with the question order randomized. The surveys were sent before winter break, during block final exams, the subsequent block’s midpoint, and after the subsequent block’s midterm examinations.

**Results:**

A total of 147 students completed the questionnaire (29.4%; n = 147/500). Most participants (73.5%) reported experiencing symptoms of work-related burnout, and 44.2% reported symptoms of depression. Client-related burnout and PHQ-9 scores were strongly correlated (Spearman r = 0.645, p < 0.001). Work-related burnout was also strongly related to client-related burnout (r = 0.739, p < 0.001) and PHQ-9 scores (r = 0.786, p < 0.001). No statistically significant differences in depression or burnout were noted throughout all four survey rounds.

**Conclusions:**

Burnout and depression severity in first-year medical students remain statistically similar throughout the academic year despite significant events such as exams or breaks. This may indicate that scheduling exams after breaks reduces any protective effect it may have on burnout/depression. It may also suggest that institutional changes such as a pass-fail preclinical curriculum meant to improve medical students’ mental health have not had the intended positive effect.

## Introduction

Depression and burnout among medical students have long been a matter of concern among medical educators, as both are highly prevalent and associated with adverse outcomes in this population [1–3]. Compared to the general population, medical students are more likely to present with signs of burnout, major depressive disorder, and generalized anxiety disorder [1, 4]. Furthermore certain groups, such as female medical students in the preclinical years, are even more likely to be affected by anxiety and depression than their peers [5]. Consequences of these negative psychological states can be seen in the correlation between burnout and the intention of dropping out of medical school [1]. Depression, suicidal ideation, and a low sense of personal accomplishment are also higher in medical students compared to residents and early-career attending physicians, indicating that medical school can be particularly stressful in the context of one’s medical career [6]. Interestingly, depersonalization among medical students plateaued early into medical school, while emotional exhaustion increased during clinical clerkships [7]. This may hint at clerkship-specific issues, such as power dynamics within care teams, and the increased pressure clerkship students face when it comes to juggling their responsibilities during rotations and their academic responsibilities as a medical student.

There are many factors that contribute to medical students’ mental health that medical educators can carefully analyze and modify with the intention of improving medical student outcomes [3, 8, 9]. One significant change was seen in 2022 when the United States Medical Licensing Exam (USMLE) Step 1 transitioned from being scored to being pass/fail, partly to reduce medical student stress experienced because of its substantial influence on their competitiveness for residency applications [10]. Additionally, the COVID-19 pandemic has led to many changes in the residency application process, such as the adoption of “signaling” programs in the United States and the continued use of remote interviewing internationally, a practice that offers to alleviate part of the financial burdens on medical students while also allowing them to represent themselves accurately during residency/fellowship interviews [11]. Current medical students face a different academic landscape and learning environment compared to prior generations. For example, USMLE Step 1 transitioning to a pass/fail scoring system caused this study’s institutional site to transition from a 24-month preclinical curriculum to an 18-month curriculum, significantly increasing the pace medical students must cover academic material and practice their clinical skills [12]. The effects of such substantial changes to the learning environment on mental health warrant further study due to the crucial role of learning environments in determining student well-being and satisfaction [9]. First-year US medical students (M1s) are an understudied group, with most studies being prior to the COVID-19 pandemic or in non-US contexts [13]. This study aims to contribute to the literature by quantifying the prevalence/severity of depression and burnout in first-year medical students (M1) in the context of major academic events, such as exams or breaks, at an institution that had altered its curriculum in response to USMLE Step 1 board exam becoming pass/fail.

## Methods

### Recruitment and participants

One hundred twenty-five M1 students at an allopathic medical school in California were contacted via email for this study on December 12 of 2022 and January 3, January 17, and January 31 of 2023. The five medical students on the research team were excluded from the survey to prevent any potential bias. An observational cohort design was used where four surveys were administered electronically via institutional email to participating medical students’ school emails, with each survey round of data collection occurring for up to seven days after it was sent. The surveys were sent out in four discrete rounds to collect burnout/depression data as the M1 students continued through the academic year and encountered events such as winter break, final exams, regular lessons, and midterms. The first 13 participants of each round were compensated with an electronic $5 Amazon gift card for their participation. Funding for these gift cards was provided by the medical school’s Student Scholars Presentation and Dissemination Initiative committee.

### Measures

The Copenhagen Burnout Inventory (CBI) consists of nineteen five-point Likert-scale questions grouped into three sections measuring burnout in employed individuals and validated for healthcare professionals [14–16]. The personal burnout section consists of six items that examine generalized symptoms of exhaustion [14]. The work-related burnout section consists of seven items that examine exhaustion related to an individual’s work [14]. The client-related burnout section consists of six items that examine exhaustion related to interacting/working with clients (defined as individuals respondents interact within their role as a medical student) [14]. Burnout severity is determined by rescaling responses to a 0–100 metric (the values being 0–25–50–75–100) and calculating the scores by taking the means of the items in each scale [14, 15]. Scores from 0 to 24 are considered as having no burnout, 25 to 49 are considered low, 50 to 74 are considered moderate, 75 to 99 are high, and an individual with a score of 100 is considered to experience severe burnout [14, 15, 17].

The Patient Health Questionnaire-9 (PHQ-9) is a nine-item scale that was developed and validated as a depression screening tool for primary care and non-psychiatric settings [18, 19]. Each question is rated from 0 to 3 (0, not at all; 1, several days; 2, more than half of all the days; 3, nearly every day), and results range from 0 to 27, with scores of 0 to 4 indicating minimal depression, 5 to 9 indicating mild depression, 10 to 14 indicating moderate depression, 15 to 19 indicating moderately severe depression, and 20 to 27 indicating severe depression [18, 19].

All three sections of the CBI were used. The order of the CBI and PHQ-9 questions were randomized and delivered separately. Due to privacy concerns about the stigmatization of mental health, no demographic questions, such as race or age, were included.

### Statistical analysis

Data analysis was conducted using IBM SPSS Statistics 28.0.1.0. The threshold for determining significant burnout or depression was based on respondent inventory scores (50 to 100 for the CBI, 10 to 27 for the PHQ-9) in accordance with the literature [14, 15, 18]. To test for normality, a Kolmogrov-Smirnov test was used, with normally distributed subsets of the data receiving a One-Way ANOVA with “Round” as the factor, whereas a Kruskal Wallis (via K Independent Samples) test with “Round” as the grouping variable with the range defined as minimum = 1 and maximum = 2 was conducted for non-normally distributed data subsets. Additionally, Pearson’s and Spearman’s correlations were obtained through the bivariate correlation function of SPSS. Statistical significance was defined as a P-value less than or equal to 0.05.

### Ethical considerations

The Institutional Review Board at California University of Science and Medicine approved our study (approval: HS-2022-35) on November 21, 2022. Respondents gave written consent for review and signature before starting their online surveys.

## Results

The response rates of the survey rounds were: Round 1 (43.2%, N = 54), Round 2 (28.0%, N = 35), Round 3 (20.8%, N = 26), Round 4 (25.6%, N = 32). The prevalence of moderate to severe depression in the participants ranged from 40.7% to 50% between the various survey rounds [Table 1]. Likewise, the prevalence of moderate to severe burnout ranged from 68.8% to 80.8% for personal burnout, 68.8% to 80.0% for work-related burnout, and 26.9% to 44.1% for client-related burnout [Table 2]. Before testing for statistically significant differences by round for the variables, a Kolmogorov–Smirnov test was run, which indicated that the PHQ-9 (p < 0.001), client-related burnout score (p < 0.001), and work-related burnout score (p = 0.046) are not normally distributed while the personal burnout score (p = 0.200) is normally distributed. The One-Way ANOVA found no statistically significant differences between the survey rounds in terms of personal burnout scores (p = 0.931). Likewise, the Kruskal–Wallis test found no statistically significant differences between survey rounds for the work-related burnout score (p = 0.709), client-related burnout score (p = 0.656), or the PHQ-9 score (p = 0.924). Client-related burnout and PHQ-9 scores (*r*(145) = 0.645, p < 0.001) were found to correlate by Spearman’s correlation. Work-related burnout was also found to correlate with client-related burnout (*r*(145) = 0.739, p < 0.001) and PHQ-9 scores (*r*(147) = 0.786, p < 0.001).

**Table 1:** Descriptive statistics of participant depression (PHQ-9) by survey round

Round	Mean	SD	Overall Depression Severity^a^	Cases of Significant Depression^b^ %	Cases by severity^a^				
					Minimal	Mild	Moderate	Moderately Severe	Severe
1	8.76 (N = 54)	± 6.85	Mild	N = 22 (40.7)	19	13	11	4	7
2	9.62 (N = 35)	± 7.24	Mild	N = 16 (45.7)	14	5	8	6	2
3	9.31 (N = 26)	± 6.82	Mild	N = 13 (50.0)	8	5	6	5	2
4	10.36 (N = 32)	± 8.57	Moderate	N = 14 (43.8)	11	7	2	7	5
Total	9.41 (N = 147)	± 7.29	Mild	N = 65 (44.2)	52	30	27	22	16

^a^Depression severity was by the following cutoffs: scores of 0 to 4 as minimal, 5 to 9 as mild, 10 to 14 as moderate, 15 to 19 as moderately severe, and 20 to 27 as severe depression[19]

^b^The threshold for significant depression is 10 to 27 points scored on the PHQ-9 as determined by the literature[19]

**Table 2:** Descriptive statistics of participant burnout (CBI) by survey round

Burnout Type	Round	Mean	SD	Overall burnout severity^a^	Cases of significant burnout^b^ %	Cases by severity^a^				
						No	Low	Moderate	High	Severe
Personal	1	58.51 (N = 54)	± 16.90	Moderate	N = 38 (70.4)	1	15	30	8	0
Personal	2	60.19 (N = 35)	± 18.27	Moderate	N = 25 (71.4)	1	9	18	7	0
Personal	3	61.44 (N = 26)	± 18.84	Moderate	N = 21 (80.8)	1	4	16	5	0
Personal	4	59.90 (N = 32)	± 24.12	Moderate	N = 22 (68.8)	3	7	13	7	2
Personal	Total	59.73 (N = 147)	± 19.15	Moderate	N = 106 (72.1)	6	35	77	27	2
Work related	1	60.11 (N = 54)	± 13.75	Moderate	N = 39 (72.2)	0	15	32	7	0
Work related	2	62.19 (N = 35)	± 14.72	Moderate	N = 28 (80.0)	0	7	22	6	0
Work related	3	60.74 (N = 26)	± 15.10	Moderate	N = 19 (73.1)	0	7	16	3	0
Work related	4	58.84 (N = 32)	± 20.24	Moderate	N = 22 (68.8)	2	8	14	8	0
Work related	Total	60.44 (N = 147)	± 15.70	Moderate	N = 108 (73.5)	2	37	84	24	0
Client related	1	42.70 (N = 54)	± 15.68	Low	N = 17 (31.5)	6	31	15	2	0
Client related	2	46.96 (N = 34)^c^	± 17.53	Low	N = 15 (44.1)	5	14	13	2	0
Client related	3	44.62 (N = 26)	± 18.93	Low	N = 7 (26.9)	2	17	4	3	0
Client related	4	47.37 (N = 31)^c^	± 22.42	Low	N = 10 (32.3)	6	15	5	4	1
Client related	Total	45.04 (N = 145)	± 18.22	Low	N = 49 (33.8)	19	77	37	11	1

^a^Burnout severity was determined by the following cutoffs: 0 to 24 as no burnout, 25 to 49 as low burnout, 50 to 74 as moderate burnout, 75 to 99 as high burnout, and 100 as severe burnout [15, 16]

^b^The threshold for determining significant burnout is 50 to 100 for the CBI in accordance with the literature [15, 16]

^c^One participant did not fill out this section during this round

The averaged PHQ-9 scores of the rounds are relatively close to the moderate depression severity cutoff of 10 points with rounds one through three ranging between 8.76 to 9.62 points while round four averages slightly higher at 10.36 [Table 1]. This resulted in round four being labeled as having a different depression severity despite the lack of statistically significant differences between survey rounds for PHQ-9 scores (p = 0.924). Meanwhile, the overall burnout severity between survey rounds stayed the same throughout all rounds for each burnout subscore [Table 2]. Personal and work-related burnout were moderate overall, while client-related burnout was low in participants overall.

## Discussion

A significantly higher proportion of the medical students surveyed displayed signs of depression and burnout than normal [19]. Compared to the overall prevalence of significant work-related burnout and personal burnout at 73.5% and 72.1% [Table 2], respectively, the overall prevalence of client-related burnout was much lower at 33.8%, corresponding to the preclinical curriculum's primarily academic focus. This high prevalence is concerning, but a further examination of the data shows that most participants exhibited moderate burnout, with very few manifesting severe burnout. One explanation for this distribution in the data is the mitigation of the rigorous and stressful nature of medical school through elements such as problem-based learning, which has been shown to potentially have positive effects on medical students’ mental health [2, 6]. These curricular alterations may have allowed medical students to better adjust to the intensity of medical school, resulting in fewer cases of severe burnout [2]. However, this cannot be concretely determined due to the anonymous nature of this study and the limited literature regarding this topic [2].

Compared to the 17.2% prevalence of depression in American 18–25 year olds, this study’s participants demonstrated a significantly higher prevalence of depression at 44.2% [Table 1] according to their PHQ-9 scores [18, 20]. This observation is in agreement with the literature and furthers the association between the competitiveness and rigor of medical school with increased psychological morbidities among medical students and demonstrates the need for access to mental health services from the start of medical school [2, 7].

Additionally, there is a discrepancy between the normally distributed Personal burnout score and the non-normal distribution of the PHQ-9, Client-related burnout, and Work-related burnout scores, as determined by Kolmogorov–Smirnov tests. One explanation could be the existence of pre-existing psychiatric risk factors or psychiatric diagnoses that had been exacerbated by the rigor and stressors of medical school. Likewise, the client-related and work-related distribution could reflect differing expectations regarding the rigor, culture, and pace of medical school among medical students with diverse experiences and backgrounds. Meanwhile, preclinical medical students in the same program generally face comparable levels of difficulty regarding expected academic performance, clinical knowledge, and participation in extracurriculars [8]. These relatively uniform stressors that preclinical medical students face could explain the normal distribution of the Personal burnout score with factors such as specialty interest and personality differences leading to variations in burnout subscores between medical students [21].

The lack of statistically significant differences between survey rounds in the One-Way ANOVA and Kruskal–Wallis tests is comparable to the literature and potentially indicates the static nature of stressors in preclinical medical students [7, 22]. The academic rigor and pace of medical school are much higher than the standard bachelor’s or master’s degree programs, potentially leading to increased perceived stressors and higher burnout scores in medical students compared to themselves at the undergraduate-level. On the other hand, a group of preclinical M1 students in their first semester is likely to face comparable stressors and, therefore, have comparable burnout scores to preclinical M1s in their second semester, as seen in the results of this study. The rapidly progressing negative effects medical training has on mental health, as seen in the literature, could be explained by the mechanism mentioned earlier, but future studies are needed before solid conclusions are drawn [8, 15].

The correlation analyses indicate a potential association between depression and burnout. The existence of a correlation between depression and burnout is not surprising, given that worsening depression can worsen burnout and vice-versa. Medical school is a high-stress environment, and the frequently observed phenomenon of burnout in medical students potentiating mental health crises in this vulnerable group underscores the growing need for medical schools and related institutions to invest in programs meant to improve current student mental health while also exploring possible avenues in which medical education can be improved for the sake of future medical student mental health [1]. This long-standing issue is exacerbated by multiple systemic and cultural factors embedded within medical education [23]. One such example at this study’s institution would be the challenges that the student wellness committee faces with the need to plan events that are applicable to all members of the community while also staying within a limited budget. During the timeframe of this study, the institution’s student wellness committee hosted a wellness event which involved decorating gingerbread houses that many students and community members enjoyed. However, as noted by members of the student wellness committee, the needs of each cohort vary significantly given many factors such as the general culture of a cohort, their current academic year, and what recent curricular changes they are faced with.

Future studies are needed to examine burnout rates in US medical students throughout their medical education as the USMLE grading transition has significantly changed the educational landscape in the US and the potential solutions that could improve outcomes and well-being in medical students. The current literature examining burnout in medical students from the United States primarily draws from data collected prior to the start of the COVID-19 pandemic. Although there is a study which reports a burnout prevalence of 75.6% in medical students, which is higher than historical norms in the United States, this study’s data was collected prior to the 2022 USMLE Step 1 grading transition [24]. As it currently stands, the impact of the USMLE Step 1 grading transition on medical students’ mental health is not fully understood which can present an issue for medical educators as aspects of one’s curriculum may have unintended effects on a student’s perceptions and priorities in medical school [25]. Despite these considerations, this study demonstrates a comparable burnout prevalence of work-related burnout and personal burnout at 73.5% and 72.1% to the post-COVID-19 study’s 75.6% [24]. Due to the nature of this study, its results cannot be generalized across the United States, but the comparable burnout prevalence does raise concerns that USMLE Step 1 transition to Pass/Fail has not had its intended effects on medical students’ mental health. As such, further studies on United States medical student burnout are warranted to see if the stress associated with USMLE Step 1 has actually decreased or has been shifted towards USMLE Step 2.

### Limitations

This study was limited by its low response rate and small sample size, as well as the inability to track respondents over time. Unfortunately, student email accounts tend to receive many emails from interest and research groups which causes email fatigue and low response rates, the limited funding did not allow for the use of more substantial incentives and the researchers were prohibited from tracking respondents out of fear the information could be used for retaliation against those who reported burnout. Out of 125 potential participants, the 1st and 3rd surveys had response rates of 43.2% (n = 54) and 20.8% (n = 26) respectively. These response rates are comparable to or slightly lower than other studies in the literature. Still, there exists a concern for selection bias since severely burnt-out or overwhelmed medical students may have chosen not to participate in the study [4]. Another limitation was the narrow timeframe that the survey rounds were spread across. Other studies in the literature examine burnout rates in medical students with annual survey rounds, but this study was conducted over the course of two months to examine how acute events (winter break, final exams, and midterm exams) affect medical student burnout [7]. As such, a latent burnout effect might have occurred, which this study may not have detected. The rapid administration of this study’s surveys could have also contributed to the variability in response rates through survey fatigue. Additionally, further data collection across a longer period of time could have been useful in order to ascertain the longer-term effect of the recent curricular changes.

Additionally, due to the sensitivity and stigmatization of mental health information, no demographic information for participant responses was recorded. This limits the generalizability of the data and the number of statistical tests that could be run to examine potential burnout risk factors. However, the researchers concluded that this was necessary to ensure honest responses from a significant portion of the participant pool due to the institution's particular circumstances at the time of this study’s conceptualization. Furthermore, this study took place in late 2022 and early 2023, which was during the COVID-19 pandemic but no specific data was collected or analysis conducted on the effect of COVID-19 on student responses. However, by this point the medical school at which this study took place had fully returned to in-person instruction and lifted all pandemic restrictions and M1 students have no exposure to hospital work where they could come into contact with COVID-19 patients. Due to these factors it was judged that the lingering effects of the COVID-19 pandemic on the medical student population were not significant enough to merit adding additional questions which would lengthen the surveys sent and possibly worsen our response rates even further.

This study used the Copenhagen Burnout Inventory, a free-to-use inventory designed to measure burnout in working adults. Since this inventory was not initially designed for medical students in particular, it is difficult to generalize the findings of this study to the rest of the literature since variations between burnout measures exist, though the CBI does correlate with the Maslach Burnout Inventory well [15, 16]. This was deemed acceptable during the design of this study as the CBI is a valid measure that could be administered with the study’s limited resources.

## Conclusions

Due to the importance placed on anonymizing the data, it is difficult to draw any conclusions regarding risk or causal factors and depression/burnout in medical students. However, the high prevalence of symptoms of depression and burnout in M1 students does indicate that medical school is a highly rigorous and stressful environment from the very beginning. Despite recent measures being taken to lower medical student anxiety, such as the transition of the USMLE Step 1 board exam to pass/fail, the intensity of medical school remains exceptional. For instance, it was initially hypothesized that round 2 would have lower rates of depression and burnout than round 1 due to the perception that a break from school would allow more time to relax and promote better mental health. However, this was not the case, as rates of depression increased from 40.7% to 45.7% and all burnout subscales also increased between Round 1 and Round 2. A possible cause of this is that the break was immediately followed by exam week, which might have increased depression and burnout and could have prevented rest and relaxation during the break. This unforeseen consequence further emphasizes the need for individual institutions to adopt student wellness policies aimed at destigmatizing struggles with mental health while allowing medical students to maintain a satisfactory work-life balance. Medical education is a very complex system that has been rapidly changing in recent years, and medical students currently undergoing training are at high risk of burning out or developing anxiety/depression while trying to adapt to the ever-shifting demands placed on them by their preceptors, professors, and themselves. Further studies into the effectiveness of current student wellness policies should be conducted as this is an aspect of medical school that many students feel can be improved [3]. In particular, methods of destigmatizing mental health issues among medical students could be a fruitful subject of research as it would improve the utilization of existing services and programs.

## Data Availability

Data Accessibility Statement: The data used to support our conclusions was not collected from any larger data set and is available in its entirety at: https://www.openicpsr.org/openicpsr/project/202723/version/V1/view.
